# Correction: Shin, S.-H.; Baek, O.-J. A Study on Internet News for Patient Safety Campaigns: Focusing on Text Network Analysis and Topic Modeling. *Healthcare* 2024, *12*, 1914

**DOI:** 10.3390/healthcare12222267

**Published:** 2024-11-14

**Authors:** Sun-Hwa Shin, On-Jeon Baek

**Affiliations:** Nursing Department, College of Nursing, Sahmyook University, Seoul 01795, Republic of Korea; shinsh@syu.ac.kr

## Error in Figure

In the original publication [1], there was a mistake in Figure 2 as published. In Figure 2, images 2-2 and 2-3 are duplicates. The corrected version of Figure 2 appears below. The authors state that the scientific conclusions are unaffected. This correction was approved by the Academic Editor. The original publication has also been updated.

## Figures and Tables

**Figure 2:**
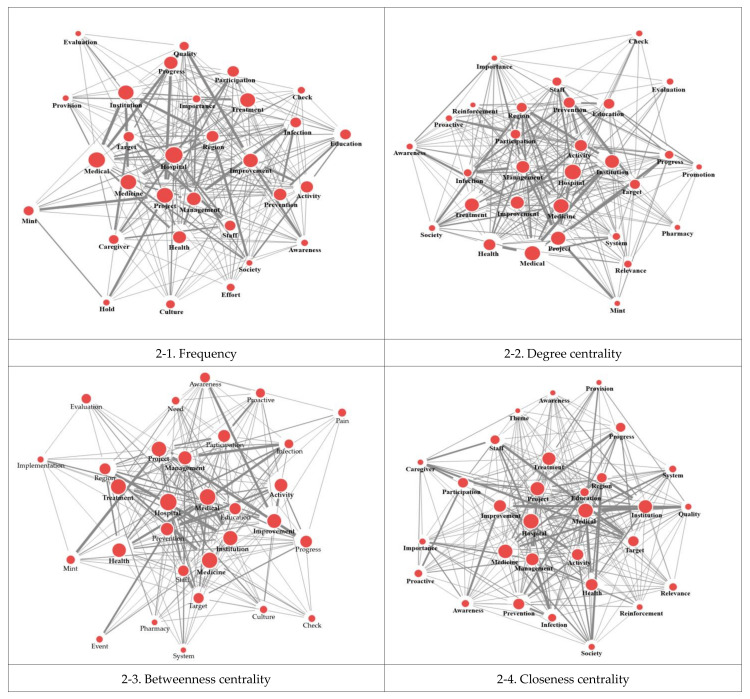
Spring network map of centrality.
